# Supplementary material for: Association between Functional Parameters of Coagulation and Conventional Coagulation Tests in the Setting of Fluid Resuscitation with Balanced Crystalloid or Gelatine: A Secondary Analysis of an In Vivo Prospective Randomized Crossover Study
Source: J Clin Med. 2022 Jul 14;11(14):4065. doi: 10.3390/jcm11144065 (PMC9316976; doi:10.3390/jcm11144065)
Supplement: Supplementary file 1 [file jcm-11-04065-s001.zip › jcm-1795013-supplementary.pdf]

Table S1. Multiple regression analysis for association of EXTEM and INTEM clotting time and standard laboratory tests, also after adjustment for haemodilution [1].

Blood sample (n=25)	EXTEM clotting time				
	**PT**	**Fibrinogen**	**PLT**	**%Haemodilution**	**'p' for the model**
Post-dilution_Optilyte	5.33±2.07 p=0.018	-0.09±0.05 p=0.1	-0.003±0.04 p=0.9	0.02±0.52 p=0.9	0.066
Post-dilution_Geloplasma	2.04±1.39 p=0.2	-0.02±0.04 p=0.5	0.008±0.03 p=0.8	-0.05±0.33 p=0.9	0.6
**Blood sample (n=25)**	**INTEM clotting time**				
	**APPT**	**Fibrinogen**	**PLT**	**%Haemodilution**	**'p' for the model**
Post-dilution_Optilyte	3.52±0.73 p=0.0001	-0.009±0.09 p=0.9	0.05±0.07 p=0.4	-0.29±0.79 p0.7	0.002
Post-dilution_Geloplasma	3.73±0.77 p=0.0001	-0.08±0.12 p=0.5	-0.005±0.08 p=0.9	-1.11±0.90 p=0.2	0.0006

[1] Values are coefficients of regression ± standard deviations, and their 'p' values, N/A – not applicable. Abbreviations: EXTEM, extrinsic pathway ROTEM assay; INTEM, intrinsic pathway ROTEM assay; APTT, activated partial thromboplastin time; Fibrinogen, fibrinogen concentration; %Haemodilution, the degree of haemodilution; PLT, platelet count; PT, prothrombin time; post-dilution_Optilyte, correlation for coagulation tests values post crystalloid infusion; post-dilution_Geloplasma, correlation for coagulation tests values post colloid infusion.

Table S2. Multiple regression analysis for association of EXTEM and INTEM alpha angle and standard laboratory tests, also after adjustment for haemodilution [1].

**Blood sample (n=25)**	**EXTEM alpha angle**			
	**Fibrinogen**	**PLT**	**%Haemodilution**	**'p' for the model**
Post-dilution_Optilyte	0.11±0.02 p<0.0001	0.04±0.01 p=0.005	-0.12±0.15 p=0.4	<0.0001
Post-dilution_Geloplasma	0.11±0.02 p=0.0002	0.05±0.02 p=0.02	0.22±0.20 p=0.3	0.0003
**Blood sample (n=25)**	**INTEM alpha angle**			
	**Fibrinogen**	**PLT**	**%Haemodilution**	**'p' for the model**
Post-dilution_Optilyte	0.03±0.01 p=0.011	0.03±0.01 p=0.004	-0.08±0.12 p=0.5	0.001
Post-dilution_Geloplasma	0.05±0.02 p=0.011	0.04±0.01 p=0.012	-0.05±0.16 p=0.8	0.005

[1] Values are coefficients of regression ± standard deviations, and their 'p' values, N/A – not applicable. Abbreviations: EXTEM, extrinsic pathway ROTEM assay; INTEM, intrinsic pathway ROTEM assay; Fibrinogen, fibrinogen concentration; %Haemodilution, the degree of haemodilution; PLT, platelet count; post-dilution_Optilyte, correlation for coagulation tests values post crystalloid infusion; post-dilution_Geloplasma, correlation for coagulation tests values post colloid infusion.

Table S3. Multiple regression analysis for association of EXTEM and INTEM clot formation time and standard laboratory tests, also after adjustment for haemodilution [1].

Blood sample (n=25)	EXTEM clot formation time			
	**Fibrinogen**	**PLT**	**%Haemodilution**	**'p' for the model**
Post-dilution_Optilyte	-0.44±0.08 p<0.0001	-0.22±0.07 p=0.003	1.10±0.80 p=0.2	<0.0001
Post-dilution_Geloplasma	-0.55±0.13 p=0.0004	-0.28±0.10 p=0.012	-1.21±1.14 p=0.3	0.0004
**Blood sample (n=25)**	**INTEM clot formation time**			
	**Fibrinogen**	**PLT**	**%Haemodilution**	**'p' for the model**
Post-dilution_Optilyte	-0.20±0.06 p=0.004	-0.18±0.05 p=0.001	0.39±0.58 p=0.5	0.0003
Post-dilution_Geloplasma	-0.29±0.10 p=0.011	-0.23±0.08 p=0.008	0.38±0.89 p=0.7	0.003

[1] Values are coefficients of regression ± standard deviations, and their 'p' values, N/A – not applicable. Abbreviations: EXTEM, extrinsic pathway ROTEM assay; INTEM, intrinsic pathway ROTEM assay; Fibrinogen, fibrinogen concentration; %Haemodilution, the degree of haemodilution; PLT, platelet count; post-dilution_Optilyte, correlation for coagulation tests values post crystalloid infusion; post-dilution_Geloplasma, correlation for coagulation tests values post colloid infusion.

Table S4. Multiple regression analysis for association of EXTEM and INTEM 10th minute clot amplitude and standard laboratory tests, also after adjustment for haemodilution [1].

**Blood sample (n=25)**	**EXTEM amplitude 10min**				
	**Fibrinogen**	**PLT**	**D-dimers**	**%Haemodilution**	**'p' for the model**
Post-dilution_Optilyte	0.13±0.02 p<0.0001	0.06±0.01 p=0.0003	-0.01±0.004 p=0.2	-0.04±0.17 p=0.8	<0.0001
Post-dilution_Geloplasma	0.12±0.02 p=0.0001	0.07±0.02 p=0.004	0.004±0.003 p=0.1	0.31±0.21 p=0.2	0.0002
**Blood sample (n=25)**	**INTEM amplitude 10min**				
	**Fibrinogen**	**PLT**	**D-dimers**	**%Haemodilution**	**'p' for the model**
Post-dilution_Optilyte	0.07±0.02 p=0.001	0.05±0.01 p=0.0005	-0.001±0.004 p=0.9	-0.03±0.15 p=0.8	0.0001
Post-dilution_Geloplasma	0.10±0.02 p=0.0001	0.07±0.02 p=0.003	0.005±0.003 p=0.1	0.05±0.17 p=0.8	0.0003

[1] Values are coefficients of regression ± standard deviations, and their 'p' values, N/A – not applicable. Abbreviations: EXTEM, extrinsic pathway ROTEM assay; INTEM, intrinsic pathway ROTEM assay; D-dimer concentration; Fibrinogen, fibrinogen concentration; %Haemodilution, the degree of haemodilution; PLT, platelet count; post-dilution_Optilyte, correlation for coagulation tests values post crystalloid infusion; post-dilution_Geloplasma, correlation for coagulation tests values post colloid infusion.

Table S5. Multiple regression analysis for association of EXTEM and INTEM maximum clot firmness and standard laboratory tests, also after adjustment for haemodilution [1].

**Blood sample (n=25)**	**EXTEM maximum clot firmness**				
	**Fibrinogen**	**PLT**	**D-dimers**	**%Haemodilution**	**'p' for the model**
Post-dilution_Optilyte	0.11±0.02 p=0.0002	0.06±0.02 p=0.001	-0.004±0.005 p=0.5	-0.02±0.19 p=0.9	0.0001
Post-dilution_Geloplasma	0.10±0.02 p=0.0004	0.07±0.02 p=0.006	0.005±0.003 p=0.1	0.38±0.21 p=0.1	0.0009
**Blood sample (n=25)**	**INTEM maximum clot firmness**				
	**Fibrinogen**	**PLT**	**D-dimers**	**%Haemodilution**	**'p' for the model**
Post-dilution_Optilyte	0.08±0.02 p=0.0008	0.05±0.01 p=0.0008	-0.0003±0.005 p=0.9	-0.04±0.16 p=0.8	0.0001
Post-dilution_Geloplasma	0.09±0.02 p=0.0002	0.07±0.02 p=0.002	0.007±0.003 p=0.016	0.14±0.18 p=0.4	0.0005

[1] Values are coefficients of regression ± standard deviations, and their 'p' values, N/A – not applicable. Abbreviations: EXTEM, extrinsic pathway ROTEM assay; INTEM, intrinsic pathway ROTEM assay; D-dimer concentration; Fibrinogen, fibrinogen concentration; %Haemodilution, the degree of haemodilution; PLT, platelet count; post-dilution_Optilyte, correlation for coagulation tests values post crystalloid infusion; post-dilution_Geloplasma, correlation for coagulation tests values post colloid infusion.
